# Human protective response induced by meningococcus B vaccine is mediated by the synergy of multiple bactericidal epitopes

**DOI:** 10.1038/s41598-018-22057-7

**Published:** 2018-02-27

**Authors:** M. Giuliani, E. Bartolini, B. Galli, L. Santini, P. Lo Surdo, F. Buricchi, M. Bruttini, B. Benucci, N. Pacchiani, L. Alleri, D. Donnarumma, W. Pansegrau, I. Peschiera, I. Ferlenghi, R. Cozzi, N. Norais, M. M. Giuliani, D. Maione, M. Pizza, R. Rappuoli, O. Finco, V. Masignani

**Affiliations:** 1 GSK, Siena, Italy; 2 University of Siena, Siena, Italy; 3 University of Bologna, Bologna, Italy

## Abstract

4CMenB is the first broad coverage vaccine for the prevention of invasive meningococcal disease caused by serogroup B strains. To gain a comprehensive picture of the antibody response induced upon 4CMenB vaccination and to obtain relevant translational information directly from human studies, we have isolated a panel of human monoclonal antibodies from adult vaccinees. Based on the Ig-gene sequence of the variable region, 37 antigen-specific monoclonal antibodies were identified and produced as recombinant Fab fragments, and a subset also produced as full length recombinant IgG1 and functionally characterized. We found that the monoclonal antibodies were cross-reactive against different antigen variants and recognized multiple epitopes on each of the antigens. Interestingly, synergy between antibodies targeting different epitopes enhanced the potency of the bactericidal response. This work represents the first extensive characterization of monoclonal antibodies generated in humans upon 4CMenB immunization and contributes to further unraveling the immunological and functional properties of the vaccine antigens. Moreover, understanding the mechanistic nature of protection induced by vaccination paves the way to more rational vaccine design and implementation.

## Introduction

*Neisseria meningitidis* is the leading cause of bacterial meningitis and sepsis worldwide, and has the highest incidence in infants and adolescents^1^. Glycoconjugated vaccines that target the polysaccharide capsule are available to protect against disease caused by meningococcal serogroups A, C, W and Y. More recently, two protein-based vaccines (4CMenB and rLP2086) have been developed against serogroup B meningococcus. 4CMenB is currently licensed in Europe, Australia, Canada and several countries of Latin America to prevent meningococcal infection in subjects of 2 months and older, and in the US for 10–25 years old subjects^2^. Recently, it has also been introduced in the UK for mass vaccination of infants, showing promising effectiveness^3^. 4CMenB is a multicomponent vaccine formulation that includes three recombinant protein antigens (NadA, NHBA and fHbp) plus detergent-extracted outer membrane vesicles (OMV) obtained from the epidemic New Zealand strain^4,5^.

Several studies have contributed to elucidate the functional roles of the three recombinant antigen components in Neisseria pathogenesis. Neisserial adhesin A (NadA) is a trimeric coiled-coil autotransporter that is involved in adhesion and invasion of human epithelial cells; the *nadA* gene is not present in all meningococcal strains, and its presence is mainly associated with hypervirulent clonal complexes^6,7^. When present, NadA can be classified in two groups, each comprising three variants: vars 1, 2 and 3 (the latter being the 4CMenB vaccine variant), are the variants most frequently associated to pathogenic strains, whereas variants 4, 5 and 6 are found predominantly in carrier isolates^6,8,9^. Cross-protection is observed among variants in the same group, but not across groups^4,10^. Neisseria Heparin Binding Antigen (NHBA) has been shown to bind heparin and heparan sulfate structures thus increasing bacterial serum resistance and contributing to epithelial cell binding^11^; the *nhbA* gene is present in all meningococcal strains and classified in many different peptide variants, mainly cross protective. Factor H binding protein (fHbp) binds human complement factor H (FH), which is a negative regulator of the alternative complement pathway^12,13^ directed to the bacterial surface, thus enabling the meningococcus to evade alternative complement-mediated killing by the host innate immune system and to survive in human serum and blood. fHbp is present in the vast majority of circulating meningococcal strains and is classified in three main variant groups; the variant group 1, also referred to as “subfamily B” and the variant groups 2 and 3 also referred to as “subfamily A”. No cross protection exists between the two subfamilies, and only limited cross protection is observed between variants 2 and 3 of subfamily A^14,15^.

Three-dimensional structures have been obtained for both NadA and fHbp proteins. NadA is a trimer composed by a membrane anchor region, a stalk domain with an extended trimeric coiled-coil fold, and a distal N-terminal head domain characterized by the presence of short wing-like structures^16^. fHbp is composed by a relatively conserved N-terminal taco-shaped beta barrel and by a variable C-terminal eight stranded beta barrel domain^17–19^; the crystal structure of the fHbp:FH complex has revealed that binding of human FH engages both fHbp protein domains^20^. Finally, the structure of the distal C-terminal region of NHBA has been solved by nuclear magnetic resonance (NMR) revealing an eight stranded beta barrel domain^21^.

Clinical studies have shown the potential of each of the three recombinant antigens to induce a protective immune response in humans against a variety of meningococcal strains; however the protein regions mostly implicated in the elicitation of antibodies with functional bactericidal activity have not been defined yet. Here we report for the first time a comprehensive functional analysis of the antibody response elicited by 4CMenB in human vaccinees.

## Results

### Isolation of Human monoclonal antibodies

Peripheral Blood Mononuclear Cells (PBMCs) from three adult subjects immunized with the 4CMenB vaccine were collected 8 days after the second dose of vaccine. Plasmablasts were isolated as single cells and were used to obtain monoclonal antibodies (mAbs) as previously described by Beernink and co-workers^22^. A total of 204 mAbs were isolated: 51 different antibody sequence pairs (V_H_/V_L_) specific for fHbp, 25 for NHBA and 128 for NadA. The heavy chain isotypes for all 3 specificities are predominantly IgG, followed by lower frequency of IgA. Despite the small sample size, the heavy and light variable region repertoires of fHbp and NadA are complex. V_H_ regions derive from all the major V_H_ subgroups and for light chains both κ and λ V region genes are found. A lower complexity was observed for the NHBA repertoire, probably reflecting the fact that NHBA specific antibodies were isolated from only 2 out of the 3 subjects, and the clonality was lower than that of fHbp and NadA. No identical V region configurations were identified between subjects.

These data indicate that 4CMenB is able to recruit a diverse repertoire of B-cells and to drive class switching and somatic hypermutation possibly leading to higher affinity and a more efficacious protective response.

To characterize more deeply the antibody response to 4CMenB, a subset of antibody sequences specific for the three Neisserial protein antigens was selected for expression and characterization. In order to maximize the immunoglobulin sequence diversity analyzed, we considered different unique rearrangements, representing a single B cell V gene configuration made by a distinct VH-D-JH rearrangement paired with a distinct VL-JL rearrangement. From the 3 subjects, 12 unique rearrangements for fHbp binding, 7 for NHBA binding and 18 for NadA binding were selected for expression as recombinant Fab fragments (Table 1).

**Table 1:** Gene usage of monoclonal antibodies isolated against fHbp, NadA and NHBA.

Antigen	Seq ID	Heavy chain					Light chain				Subj
		V	D	J	IgCH	% Identity to VH GL	V	J	IgCL	% Identity to VL GL	
**fHbp**	**1A12**	IGHV5-51*01	IGHD6-19*01	IGHJ5-1*02	IgG	88.89	IGKV1-39*01	IGKJ2-1*01	Igk	92.42	**A**
	**1A3**	IGHV3-23*04	IGHD1-26*01	IGHJ6-1*02	IgG	96.53	IGKV3-20*01	IGKJ2-1*04	Igk	98.50	
	**2A1**	IGHV3-23*04	IGHD3-10*01	IGHJ4-1*02	IgG	90.63	IGKV3-11*01	IGKJ4-1*01	Igk	95.83	
	**10G7**	IGHV3-23*01	IGHD3-22*01	IGHJ5-1*02	IgG	92.71	IGKV3-11*01	IGKJ4-1*01	Igk	95.83	
	**1G3**	IGHV3-30*04	IGHD6-19*01	IGHJ4-1*02	IgG	94.79	IGKV3-11*01	IGKJ3-1*01	Igk	96.21	**B**
	**2D2**	IGHV4-b*02	IGHD4-4*01	IGHJ4-1*02	IgG	94.44	IGKV3-20*01	IGKJ3-1*01	Igk	98.13	
	**3H8**	IGHV3-11*01	IGHD5-12*01	IGHJ4-1*02	IgA	88.89	IGLV2-11*01	IGLJ2-1*01	Igλ	90.00	
	**4F7**	IGHV3-9*01	IGHD5-5*01	IGHJ4-1*02	IgG	92.36	IGLV3-21*02	IGLJ1-1*01	Igλ	96.55	
	**7B10**	IGHV3-23*04	IGHD2-2*01	IGHJ4-1*02	IgG	91.64	IGKV3-20*01	IGKJ5-1*01	Igk	96.25	**C**
	**4A9**	IGHV4-30.4*01	IGHD3-3*01	IGHJ4-1*02	IgG	92.10	IGKV3-15*01	IGKJ2-1*01	Igk	98.80	
	**3F10**	IGHV1-46*01	IGHD4-23*01	IGHJ2-1*01	IgG	90.49	IGLV1-40*01	IGLJ3-1*02	Igλ	94.44	
	**2C1**	IGHV3-30*04	IGHD2-21*01	IGHJ4-1*02	IgG	92.71	IGLV3-1*01	IGLJ2-1*01	Igλ	93.87	
**NHBA**	**10C3**	IGHV1-18*04	IGHD2-15*01	IGHJ4-1*02	IgG	94.44	IGLV2-18*01	IGLJ3-1*02	Igλ	98.89	**A**
	**3A5**	IGHV3-33*01	IGHD3-3*01	IGHJ5-1*02	IgG	97.56	IGLV1-51*01	IGLJ2-1*01	Igλ	98.50	
	**12E1**	IGHV1-3*01	IGHD3C-22*01	IGHJ5-1*02	IgG	89.24	IGKV2-30*01	IGKJ5-1*01	Igk	95.64	
	**2H1**	IGHV4-61*03	IGHD1-26*01	IGHJ4-1*02	IgG	95.88	IGKV1-39*01	IGKJ2-1*01	Igk	96.95	**C**
	**3D8**	IGHV4-61*01	IGHD6-6*01	IGHJ4-1*02	IgG	94.50	IGKV3-20*01	IGKJ5-1*01	Igk	92.13	
	**5H2**	IGHV4-61*01	IGHD6-6*01	IGHJ4-1*02	IgG	95.19	IGKV1-39*01	IGKJ2-1*01	Igk	95.70	
	**4D11**	IGHV4-61*01	IGHD3-9*01	IGHJ4-1*02	IgG	95.53	IGKV1-39*01	IGKJ1-1*01	Igk	96.59	
**NadA**	**11F7**	IGHV3-7*03	IGHD7C-27*01	IGHJ4-1*02	IgG	94.79	IGLV3-27*01	IGLJ2-1*01	Igλ	94.64	**A**
	**5D11**	IGHV1-3*01	IGHD6-6*01	IGHJ4-1*02	IgA	87.37	IGKV3-20*01	IGKJ3-1*01	Igk	93.73	
	**12H11**	IGHV1-3*01	IGHD1C-20*01	IGHJ4-1*02	IgG	91.67	IGKV3-20*01	IGKJ1-1*01	Igk	98.50	
	**10D12**	IGHV3-15*01	IGHD3-9*01	IGHJ4-1*02	IgG	92.83	IGKV4-1*01	IGKJ5-1*01	Igk	96.10	
	**1A7**	IGHV3-23*04	IGHD3-22*01	IGHJ3-1*02	IgA	91.97	IGKV1-17*01	IGKJ3-1*01	Igk	92.05	**B**
	**2A3**	IGHV3-30*14	IGHD2-15*01	IGHJ4-1*02	IgG	90.59	IGLV3-10*01	IGLJ2-1*01	Igλ	97.32	
	**1A12**	IGHV4-34*02	IGHD5-24*01	IGHJ4-1*02	IgG	87.02	IGKV1-27*01	IGKJ1-1*01	Igk	93.56	
	**1C6**	IGHV3-48*03	IGHD6-13*01	IGHJ6-1*02	IgG	94.79	IGKV3-20*01	IGKJ4-1*01	Igk	96.63	
	**2A6**	IGHV4-b*02	IGHD2-2*01	IGHJ3-1*02	IgG	87.15	IGKV1-17*01	IGKJ4-1*01	Igk	93.25	
	**2C4**	IGHV2-5*07	IGHD5C-5*01	IGHJ5-1*01	IgG	92.44	IGKV1-12*01	IGKJ1-1*01	Igk	90.94	
	**6B8**	IGHV1-3*01	IGHD6-19*01	IGHJ6-1*02	IgG	94.44	IGKV3-11*01	IGKJ4-1*01	Igk	96.59	**C**
	**4F12**	IGHV4-30.2*01	IGHD3C-10*01	IGHJ5-1*02	IgG	96.56	IGKV1-39*01	IGKJ4-1*01	Igk	97.71	
	**1A3**	IGHV5-51*01	IGHD3-10*01	IGHJ5-1*02	IgG	94.44	IGLV3-19*01	IGLJ3-1*02	Igλ	93.87	
	**7F11**	IGHV3-23*01	IGHD4-23*01	IGHJ6-1*02	IgG	95.49	IGKV2-28*01	IGKJ4-1*01	Igk	98.89	
	**3A6**	IGHV4-30.2*01	IGHD1-26*01	IGHJ3-1*02	IgG	96.91	IGKV3-20*01	IGKJ1-1*01	Igk	96.63	
	**1E5**	IGHV3-21*01	IGHD3-10*01	IGHJ4-1*02	IgG	98.96	IGLV3-1*01	IGLJ2-1*01	Igλ	98.08	
	**1F7**	IGHV3-30*18	IGHD3-10*01	IGHJ2-1*01	IgA	91.29	IGLV10-54*01	IGLJ1-1*01	Igλ	96.25	
	**3H12**	IGHV3-33*01	IGHD3-10*01	IGHJ4-1*02	IgG	97.22	IGLV3-21*03	IGLJ2-1*01	Igλ	97.66	

The percentage of identity of derived nucleotide sequence to the inferred germline (GL) alleles for VH (V, D, J) and VL (V, J) chains is indicated for each antibody (SeqID). The alleles are reported according to the IMGT nomenclature (ref.^50^, www.imgt.org). Heavy (IgCH) and light (IgCL) chain isotype and the subject from whom the plasmablasts were isolated (Subj) are also reported.

### Biochemical characterization of the human immune response to fHbp by Fab and mAb analysis

Twelve anti-fHbp Fabs were produced in *E. coli*. An affinity ranking evaluation of the Fabs for its cognate antigen was obtained by a high-throughput method based on Gyrolab assay data^23^. This patented method (patent number WO2015014922 A3) is based on the mathematical modeling of the reaction profile between antibody and antigen obtained by Gyrolab. The mathematical modeling of the reaction profiles allows derivation of a parameter, the W score, which is related to the width of the curve and permits ranking of the antibodies according to their affinity.

The Gyrolab results indicate that most of the Fabs showed high to medium level of affinity for recombinant fHbp variant 1 (Table 2) as well as recognition of the native homologous protein on the surface of the MenB MC58 strain as measured by flow cytometry analysis (Table 2). Only one of the Fabs (2D2) failed to bind the homologous protein antigen, either in the recombinant or native form (Table 2). For epitope mapping, we initially employed the Protein chip technique that makes use of a microarray where overlapping fragments of different lengths and spanning the entire sequence of the proteins of interest are spotted^24,25^. Protein chip analysis experiments suggested that all the Fabs recognized conformational epitopes, either located on the C-terminal beta-barrel domain or present on the full-length protein, asshorter fragments were not recognized (Table 2). Cross reactivity with different fHbp variants was also assessed by the Protein chip assay, and results indicated that only two Fabs (1A12 and 1G3) recognized all the 3 main variants of the protein (Table 2).

**Table 2:** Main features of selected anti-fHbp Fabs and mAbs.

fHbp											
Fab characterization						mAb characterization					
Fabs	Protein Chip			Affinity ranking on var1^b^	Flow cytometry^c^	Cross-reactivity					
	Binding region on var1 (aa)	Cross-reactivity^a^			MC58 var1 strain	PepScan on 12 fHbp variants^d^	Flow cytometry^c^		mAb affinity on fHbp variants (K_D_ by SPR)^e^		
		var2	var3				UK104 var2 strain	UK320 var3 strain	var1	var2	var3
2D2	−	−	−	n.d.	−						
1A3*	Full length	−	−	Medium-Low	+	no binding	−	−	1.67 ± 0.39 E-10 M		
4F7	Full length	−	−	n.d.	+						
7B10*	Full length	−	−	High	+	no binding	−	−	0.93 ± 0.02 E-10 M		
4A9	Full length	+/−	+/−	n.d.	−						
1G3*	Full length	+	+	High-Medium	+	no binding	+	+	1.72 ± 0.03 E-10 M	1.25 ± 0.09 E-10 M	1.28 ± 0.60 E-10 M
3H8	Full length	+	−	Medium-Low	+						
2C1*	Beta-barrel (319–434)	−	−	High	+	no binding	−	−	0.90 ± 0.05 E-11 M		
3F10	Beta-barrel (319-434)	−	−	Medium	+						
1A12*	Beta-barrel (319–434)	+	+	High-Medium	+	no binding	+	+	1.78 ± 0.8 E-11 M	2.23 ± 0.01 E-10 M	2.10 ± 0.1 E-08 M
10G7	Beta-barrel (319–434)	−	−	Medium	+						
2A1	Beta-barrel (319–434)	+	−	High-Medium	+						

^*^Fabs selected for full-length IgG1 production.

^a^Fabs cross-reactivity evaluated by Protein chip is indicated as “+” or “−” if a positive signal or no signal was detected and as “+/−” when a low intensity signal was observed. “var” indicates protein variant.

^b^Affinity ranking was defined by GyroLab based on W score: Fabs with W scores lower than 6 were ranked as “High” affinity Fabs (K_D_ > 10E-10M), Fabs with 6 < W < 9 were ranked as “Medium” affinity (10E-10M < K_D_ < 10E-8M); Fabs with W score >9 were ranked as “Low” affinity (K_D_ > 10E-8M); “n.d.” stands for not detected.

^c^Ability of Fabs and mAbs to bind to antigen expressed on bacterial surface is indicated as “+” or “−” if a positive shift or no signal was observed by Flow cytometry analysis respectively.

^d^For mAbs characterization, linear epitopes identified by PepScan and cross-reactivity on 12 fHbp variants (1.1/1.4/1.13/1.14/1.15/2.16/2.19/2.25/2.77/3.28/3.45/NL10A) are reported when available while “no binding” indicates that no signal was detected in tested conditions;

^e^mAbs affinity to 4CMenB variant as well as to other variants for cross-reactive mAbs is reported as K_D_ derived from Surface Plasmon Resonance experiments.

Based on the results above, five antibodies were selected for expression as full length mAbs, in IgG1 format. Three of the mAbs were selected for showing medium-high affinity for the full-length protein (in the Gyrolab assay), while the remaining two mAbs were selected as they recognized specifically the fHbp C-terminal domain. and the remaining three. Further selection was operated on cross reactivity to different variants, with priority given to mAbs that were either specific for variant 1 (1A3, 7B10 and 2C1) or cross-reactive with each of the three main variants (1G3 and 1A12).

By Single Cycle Kinetics (SCK) surface plasmon resonance (SPR) experiments performed with increasing concentrations of tested antigen injected on captured mAbs, affinity constants from the high picomolar to the nanomolar range were measured for all the mAbs on the homologous variant (Table 2). Of the two cross-reactive mAbs, 1G3 showed the same affinity towards all 3 variants, whereas 1A12 had lower affinity to variant 2 and particularly variant 3, compared to variant 1. Flow cytometry results obtained with mAbs reflected those previously obtained with Fabs, confirming recognition of the native protein by all the selected mAbs. Peptide Scanning (PepScan) experiments performed using peptide microarrays failed to show binding of mAbs to any of the overlapping 15-mer peptides spanning the entire fHbp protein sequences of 12 different variants. More sensitive approaches should be applied to better define the target epitope sequences.

**Figure 1:**
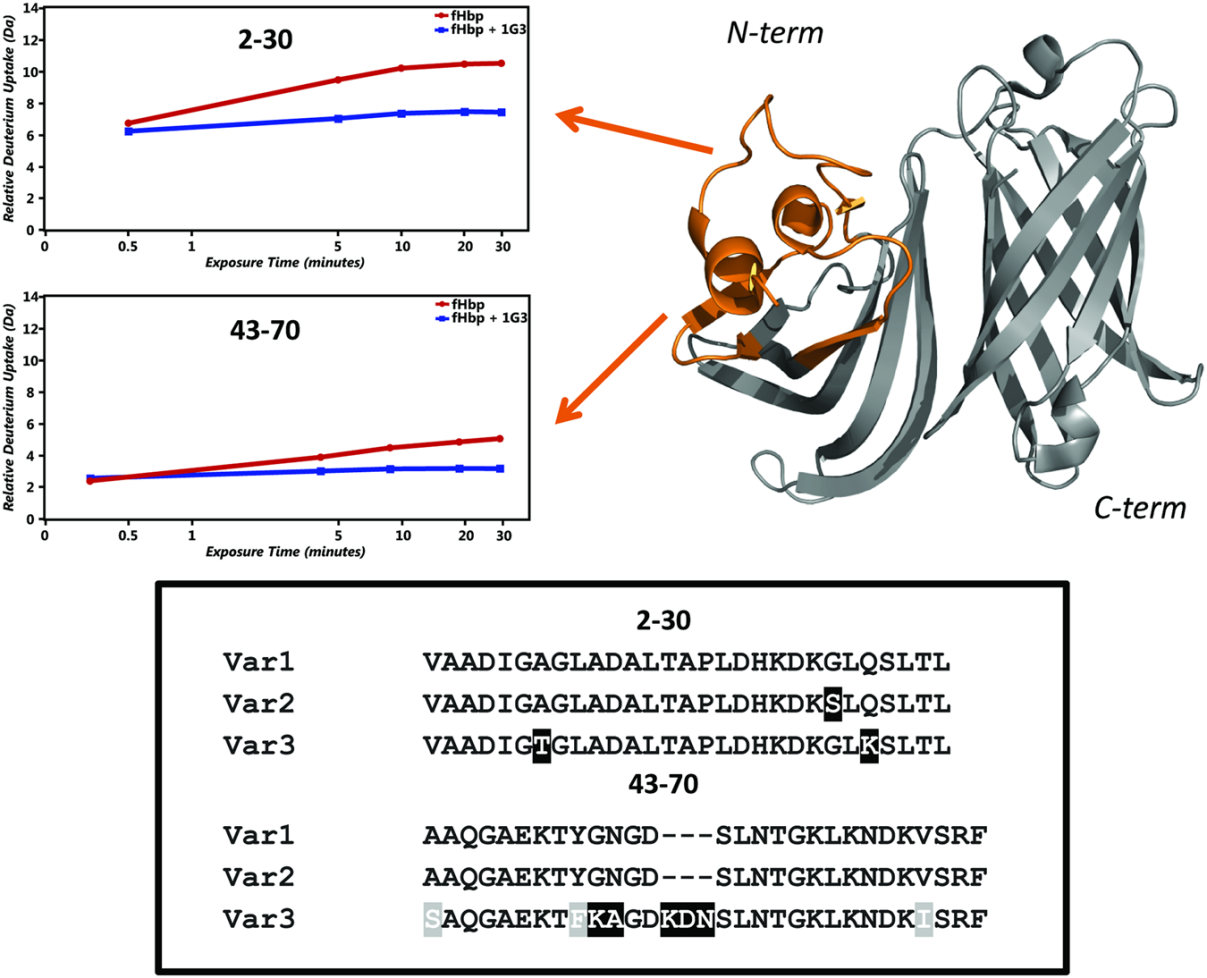
Epitope mapping by HDX-MS of the fHbp variant 1 region targeted by cross reactive mAb 1G3. (**a**) Two peptides show differential deuterium incorporation by HDX-MS, boxes show deuterium uptake over 30 minutes for fHbp peptides in the absence (red curve) or presence (blue curve) of mAb 1G3. (**b**) Multiple sequence alignment of the epitope peptides across variant 1, variant 2 and variant 3 fHbp. Residues that are conserved across all sequences are not highlighted. Residues highlighted in gray indicated conservative mutations and in black deletions and non-conservative mutations.

**Figure 2:**
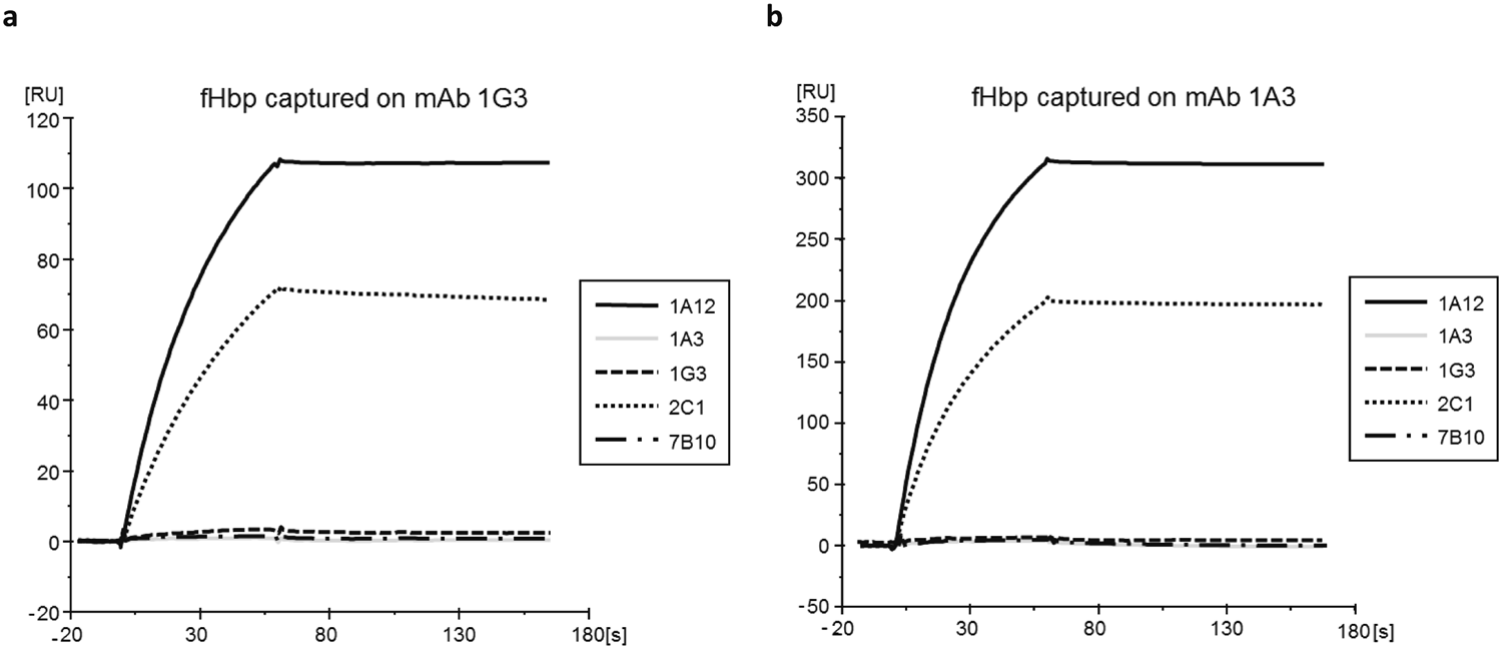
SPR Sensorgrams of various mAbs with fHbp:mAb complexes. 100 RU of fHbp were captured on immobilized mAbs 1G3 (panel a) or 1A3 (panel b). After a stabilization period, various mAbs (100 nM) were injected for 60 s and the dissociation of eventually formed ternary complexes was followed for another 120 s. The fHbp capturing phase is omitted; sensorgrams were aligned at the start of injection of the second mAb (baseline = 0 RU).

To further elucidate the recognition profile of the anti-fHbp mAbs, we applied the HDX-MS approach described previously^26^ to define the region targeted by the cross-reactive mAb 1G3, which in the Protein chip assay was only able to bind the full length protein. HDX-MS data indicate that the antibody/antigen interaction involves two non-consecutive peptides located on a well-structured region in the N terminus of fHbp (Fig. 1). Multiple sequence alignment of the three variants shows that the epitope is conserved, in agreement with the cross reactive nature of the mAb (Fig. 1). To verify whether other human mAbs targeting the full length protein could bind the same epitope as 1G3, SPR competition experiments were performed. The results show that 1A3, 7B10 and 1G3 compete for the same binding site on fHbp (Fig. 2). Therefore, these three mAbs are hereafter termed ‘anti-N-term mAbs’ whereas, as expected, 1G3 does not compete with either 1A12 or 2C1, which both target the β-barrel domain (Fig. 2).

fHbp binds human FH with high affinity^20^. Previously, we used flow cytometry analyses to show that anti-fHbp mAbs generated in mice and targeting the FH binding site were able to inhibit binding of FH to fHbp^26^. In contrast, none of the human mAbs analyzed in this study were able to inhibit the binding of fHbp to factor H, in line with data reported elsewhere using Fabs^22^ (see Supplementary Fig. S1).

### Functional characterization of anti-fHbp mAbs

**Figure 3:**
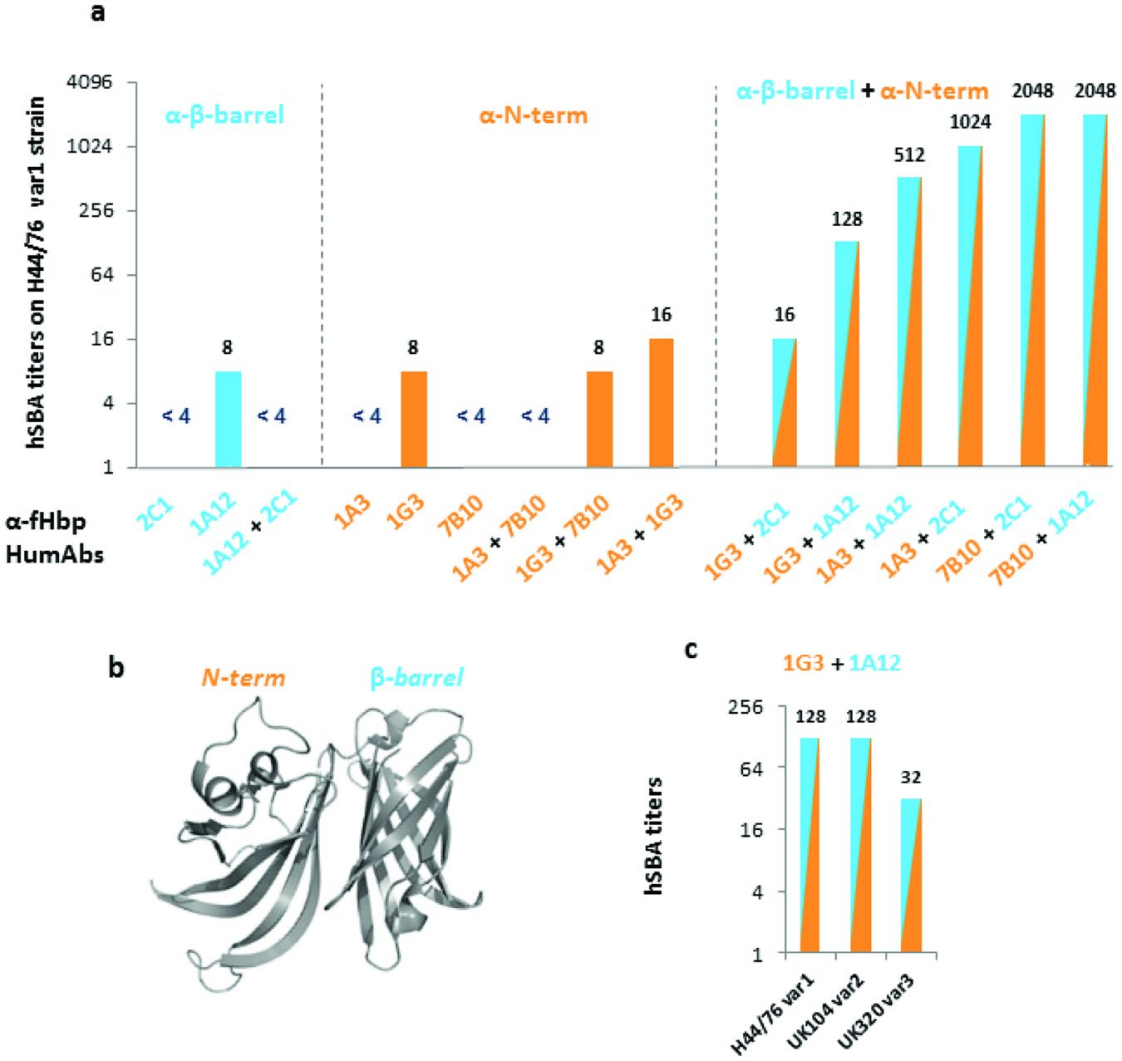
Functionality of anti-fHbp mAbs assessed by the Serum Bactericidal Activity Assay. Human serum bactericidal assay^27^ titers with combinations of mAbs targeting N-terminal (orange) and beta-barrel (blue) domains of fHbp; (**a**) localization of N-terminal and beta-barrel domain on three-dimensional structure of fHbp^19^; (**b**) hSBA titers of cooperative mAbs combination (1G3 + 1A12) on *Neisseria meningitidis* strains expressing different fHbp variants (**c**). Threshold for positive titer was defined as >4, antibody couples are considered cooperative if the titer obtained with mAbs combination is at least fourfold higher than titer obtained with single mAbs.

The functionality of the purified mAbs was then assessed by the Serum Bactericidal Activity (SBA)^27^ assay, using human serum as source of exogenous complement (Fig. 3a). When tested alone, only two mAbs showed positive hSBA titers against the serogroup B meningococcal H44/76 strain that expresses high levels of fHbp variant 1 (Fig. 3a). However, when the mAbs were tested in paired combination, they showed synergistic activity and most of the couples promoted bacterial killing (Fig. 3a). Interestingly, couples displaying a four-fold increase in hSBA titers versus mAbs tested alone are mainly composed by antibodies targeting different fHbp domains (i.e. one mAb anti-β-barrel paired with another mAb anti-N-term) (Fig. 3a).

Remarkably, the combination of the two cross-reactive mAbs 1G3 and 1A12 generated bactericidal activity against strains expressing each of the three fHbp variants (Fig. 3c). Overall, the results indicate that while mAbs tested alone failed to elicit a strong SBA response, when mAbs targeting different regions on fHbp were combined, it resulted in a synergistic effect and robust hSBA titers were obtained. Of note, synergistic activity is observed with couples of mAbs isolated either from the same (i.e. 1A3 + 1A12 and 7B10 + 2C1) or from different subjects (i.e.1A3 + 2C1 and 7B10 + 1A12).

### Biochemical characterization of the human immune response to NHBA by Fab and mAb analysis

Epitope mapping performed by Protein chip analysis revealed that four of the seven anti-NHBA Fabs bound to the NHBA C-terminal beta-barrel domain while only two recognized the N-terminal region (Table 3). One of the Fabs (3D8) did not show any appreciable binding in any of the techniques used and therefore it was not tested further. The Gyrolab assay was used to determine binding affinities of Fabs for the NHBA P2 variant, which were higher for C-terminal binders compared to N-terminal binders. Flow cytometry analysis showed that five out of the six Fabs were able to recognize the native protein expressed on the surface of the M4407 and 5/99ΔΔ CP2 MenB strains expressing the P2 variant of NHBA. The cross-reactivity of Fabs for different NHBA variants (P3, P17, P20 and P21) was also analyzed by Protein chip (Table 3). Fab 10C3 bound the P2 and P3 variants, but did not cross-react with the other three NHBA variants, that have a variable amino acid sequence in the identified target region. All the other Fabs cross-reacted with all the spotted NHBA variants, highlighting the presence of conserved epitopes (e.g. in the beta-barrel domain targeted by Fabs 4D11 and 5H2) (Table 3).

**Table 3:** Main features of selected anti-NHBA Fabs and mAbs.

NHBA								
Fab characterization						mAb characterization		
Fabs	Protein chip			Affinity ranking on P2^b^	Flow cytometry^c^	Cross-reactivity	mAb affinity on P2 (K_D_ by SPR)^e^	Flow cytometry^c^
	Binding region on P2 (aa)	Cross-reactivity^a^			(M4407 and 5/99DD P2 strains)	PepScan on 10 NHBA variants^d^		(M4407 and 5/99∆∆ CP2 strains)
		P3	P17/P20/P21			Linear epitope		
3D8	−	−	−	n.d.	n.a.			
12E1*	N-term (46–64)	+	+	n.d.	−	50-AAVS[AE]ENTGN-60	0.95 ± 0.22 E-10M	+
10C3*	N-term (149–275)	+	−	Low	+	no binding	2.12 ± 0.5 E-08 M	+
2H1	C-term (351–466)	+	+	High	+			
3A5	C-term (351–466)	+	+	High	+			
4D11*	C-term (351–466)	+	+	High	+	no binding	2.14 ± 0.17 E-10 M	+
5H2*	C-term (351–466)	+	+	High-Medium	+	no binding	1.01 ± 0.01 E-09 M	+

*^,a,b,c,e^See Table 2.

^d^For mAbs characterization, linear epitopes identified by PepScan on 10 NHBA variants (P1/P2/P3/P5/P10/P17/P18/P20/P21/P29) are reported when available while “no binding” indicates that no signal was detected in tested.

“n.a.”: not available.

Four anti-NHBA antibodies were selected for expression as full length mAbs (IgG1 format) (Table 3), including both the N-terminal-targeting antibodies (12H1 and 10C3) and two beta-barrel domain binders (5H2 and 4D11) showing medium and high affinity, respectively. In line with the Gyrolab results, affinity constants calculated by SPR on the NHBA vaccine variant were higher for the two mAbs targeting conserved epitopes at the C-term, whereas K_D_ was higher for mAb 10C3 (Table 3). The four selected mAbs were tested for their recognition profile by Protein chip experiments on a set of overlapping NHBA fragments derived from the P2 vaccine variant, confirming the results obtained with the corresponding Fabs (Table 3).

The HDX-MS approach was applied to study the interaction of NHBA and mAb 10C3, and confirmed that the antibody targets the same region identified by protein array (see Supplementary Fig. S2). Interestingly, Pep Scan and Protein chip experiments highlighted that only mAb 12E1 recognized a linear and well conserved epitope on NHBA (P1, P2, P3, P5, P10, P17, P18, P20, P21 and P29) (Table 3).

### Functional characterization of anti-NHBA mAbs

**Figure 4:**
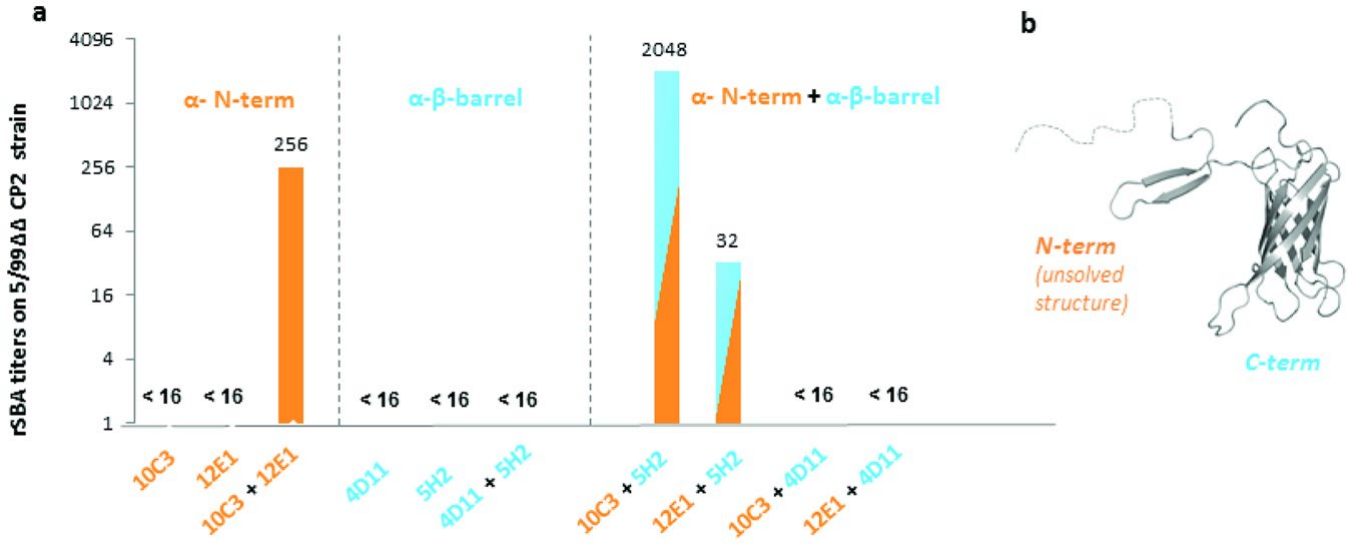
Functionality of anti-NHBA mAbs assessed by the Serum Bactericidal Activity Assay. Rabbit serum bactericidal assay (rSBA) titers obtained with combinations of mAbs targeting the N-terminal (orange) or beta-barrel (blue) of NHBA. (**a**) Three-dimensional structure of the C-term domain of NHBA^21^. Structural data are not yet available for the N-terminal domain. (**b**) Threshold for positive titer was defined as >16, antibody couples are considered cooperative if the titer obtained with mAbs combination is at least fourfold higher than titer obtained with single mAbs.

When tested in hSBA against strains carrying the homologous antigen, none of the human Abs elicited positive titers, neither alone nor in combination with a second mAb. This result was not unexpected, as anti-NHBA antisera were previously shown to confer protection against bacteremia in the absence of bactericidal activity when using human serum as source of exogenous complement^28^. In contrast, when baby rabbit serum was used as source of exogenous complement, positive titers were generated with the combination of mAbs 10C3 + 5H2, which were isolated from different subjects and targeted the N-term and the C-term portions of the protein, respectively (Fig. 4a). Interestingly, also the mAbs 10C3 and 12E1 showed a synergistic effect leading to positive bactericidal titers when used in combination. These two mAbs were isolated from the same subject and have been shown to target non-overlapping epitopes on the N-term of NHBA (Table 3).

### Biochemical characterization of the human immune response to NadA by Fab and mAb analysis

Of the 18 Fabs against NadA variant 3, by Protein chip analysis eight bound the same epitope in the head portion of NadA variant 3 (aa 52–75), two Fabs recognized the neck-stalk domain, while the remaining eight Fabs bound different epitopes along the stalk region (Table 4). Affinity characterization by Gyrolab assay revealed that all the Fabs targeting the stalk region displayed high or medium-high affinity, whereas the majority of the low-medium affinity binders recognized the head domain (Table 4). In flow cytometry analysis, twelve of the eighteen Fabs were able to recognize the native proteins on the surface of MenB strain NMB, which expresses the homologous vaccine variant (Table 4). Interestingly, three of the flow cytometry negative Fabs target the C-terminal distal region of the stalk, which is presumably very close to the membrane in the natively expressed antigen and regarding the other three, one recognized the central part of the stalk, two map on the head (Table 4).

**Table 4:** Main features of selected anti-NadA Fabs and mAbs.

NadA									
Fab characterization				mAb characterization					
Fabs	Protein Chip	Affinity ranking on var3^b^	Flow cytometry (NMB var3 strain)^c^	Cross-reactivity					mAb affinity on var3 (K_D_ by SPR)^e^
	Binding region on var3 (aa)			PepScan^d^			Flow cytometry^c^		
				linear epitope on var3	var1/var2	var4/var5	var1	var2	
2C4*	Head (16–107)	Low	+	no binding			+	+	2.16 ± 0.5 E-10 M
1A12*	Head (37–100)	Medium	+	no binding			+	+	n.d. (High)
5D11*	Head (52–75)	Medium	+	61-DEDGTIT-67	+	−	+	+	2.10 ± 0.03 E-11 M
12H11*	Head (52–75)	Low	+	61-DEDGTIT-67	+	−	+	+	3.25 ± 0.04 E-11 M
6B8	Head (52–75)	Medium-low	+						
11F7*	Head (52–75)	Medium	+	57-IYDIDEDGTIT-67	+	−	+	+	1.25 ± 0.03 E-10 M
1A3	Head (52–75)	High	+						
1A7	Head (52–75)	Low	+						
1F7	Head (52–75)	n.d.	−						
2A6	Head (52–75)	n.d.	−						
7F11*	Stalk (95–170)	Low	+	no binding			+	+	0.77 ± 0.05 E-11 M
1C6*	Stalk (100–170)	High	+	no binding			+	+	1.62 ± 0.36 E-11 M
10D12*	Stalk (158–182)	High-Medium	−	169-EAVADTV-175	+	−	+	+	4.94 ± 1.30 E-10 M
10E8	Stalk (158–192)	High	+						
3H12*	Stalk (219–275)	High	+	no binding			+	+	n.d. (High)
2A3*	Stalk (230–342)	High	−	334-LAEQ-337	+	+	−	−	1.07 ± 0.01 E-10 M
3A6*	Stalk (287–333)	High	−	no binding			−	−	n.d. (High)
4F12	Stalk (287–333)	High	−						

*^,b,c,e^See Table 2.

^d^For mAbs characterization, linear epitopes identified by PepScan on NadA 4CMenB variant are reported when available while “no binding” indicates that no signal was detected in tested conditions; cross-reactivity to other NadA variants (var1, var2, var4 and var5), identified by PepScan, is indicated as “+” or “−”.

These results above led to the selection of 11 Fabs to be expressed as IgG1 (Table 4). Eight of the mAbs were selected as they recognized different epitopes along the neck and the stalk domains, while the remaining three mAbs all bound to the same head portion but with different affinities. The affinity for the recombinant NadA variant 3, the 4CMenB variant, was determined by SPR and was found to be in the high picomolar range and comparable among all mAbs (Table 4). The Protein chip analysis confirmed the epitopes previously identified with the corresponding Fabs, in particular 5 out of 11 bind the head domain of NadA, while 6 out 11 the stalk domain (Table 4). Remarkably, the PepScan data of five mAbs (11F7, 5D11, 12H11, 10D12 and 2A3) further defined the target sequence, highlighting two partially overlapping linear epitopes (57 - IYDI[GD]EDGTIT- 67 and 61 - [GD]EDGTIT -67) in the head domain that are conserved in the variant 1, variant 2 and variant 3 of NadA (Table 4). Similarly, the epitope (169 - EAVADTV - 175) recognized by 10D12 is present in the stalk domain of the three variants most frequently associated to pathogenic strains. More interestingly, the short epitope targeted by 2A3 (334-LAEQ-337) is well conserved in all the five NadA variants tested, including the variants 4 and 5 frequently associated to carrier strains (Table 4). Flow cytometry analysis on strains expressing NadA variant 1 and variant 2 highlighted the cross-reactivity exerted by most of the analyzed mAbs against variant 1, variant 2 and variant 3, indicating that both linear and conformational epitopes are conserved in the three NadA variants (Table 4). Only the two mAbs (2A3 and 3A6) targeting the C-terminal distal region of the stalk were flow cytometry negative suggesting that this part of the protein natively expressed on the bacterial surface is not accessible to antibodies (Table 4).

### Functional characterization of anti-NadA mAbs

**Figure 5:**
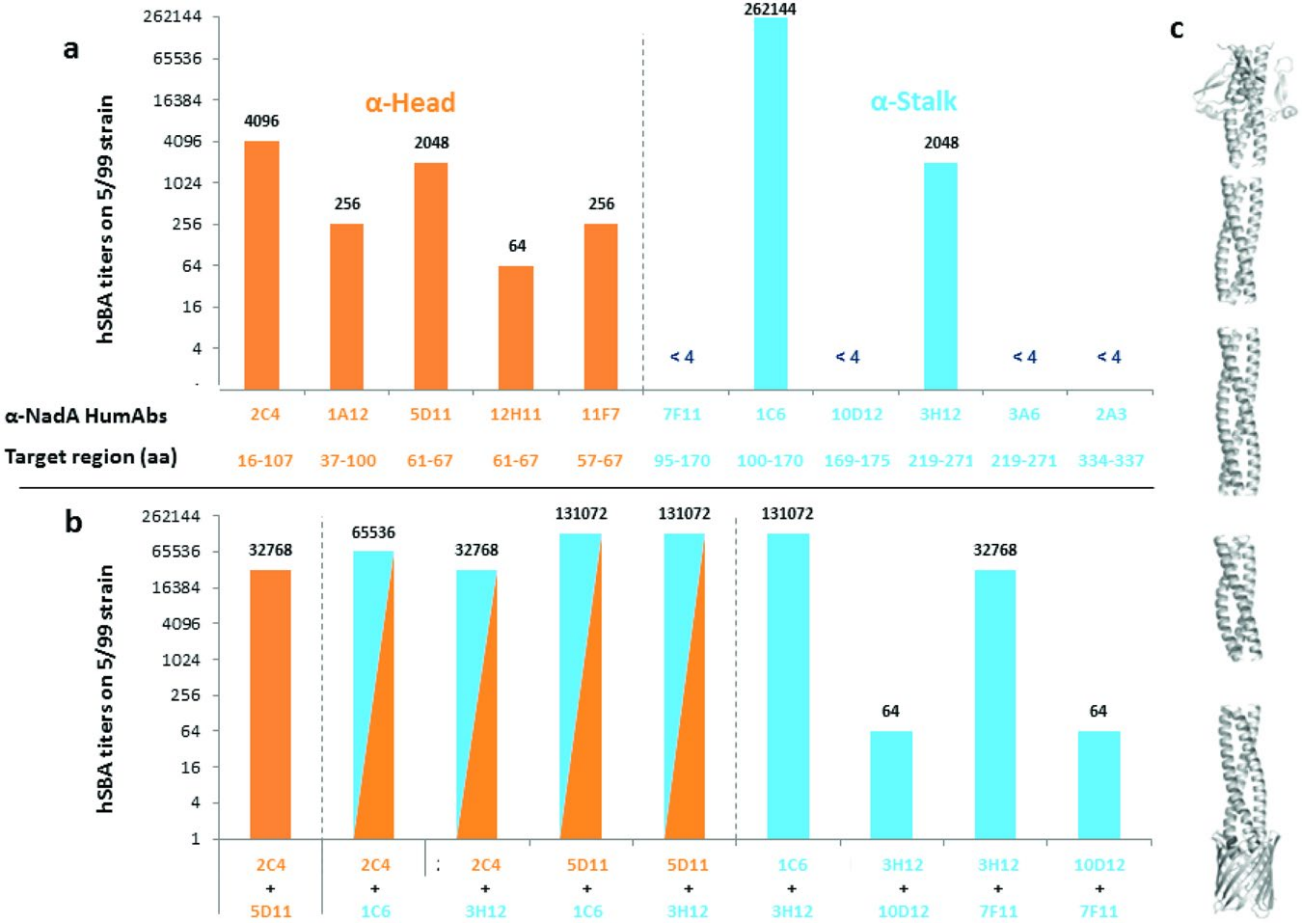
Functionality of anti-NadA mAbs assessed by the Serum Bactericidal Activity Assay Human serum bactericidal assay^27^ titers obtained with single mAbs (**a**) or combinations (**b**) targeting NadA head (orange) or stalk (blue). NadA tridimensional structure^21^. (**c**) Threshold for positive titer was defined as >4, antibody couples are considered cooperative if the titer obtained with mAbs combination is at least fourfold higher than titer obtained with single mAbs. *Coordinates of the epitope recognized by each mAb on the NadA variant 3 sequence.

hSBA analysis was performed with all the selected mAbs against the MenB 5/99 strain, which expresses high amounts of the heterologous NadA variant 2. The results indicate that all the five mAbs targeting the head domain elicit positive and comparable hSBA titers, whereas only two out of the six mAbs that bind the stalk are hSBA positive (Fig. 5a). Notably, mAb 1C6 binds an epitope located on the region 100–170 of the stalk and elicits an hSBA titer of 262,144 that is 2 logs greater than the average hSBA titers elicited by any other mAb. In contrast, mAb 7F11, which binds an overlapping epitope (95–170) is hSBA negative (Fig. 5a). A significant synergistic activity was observed when two anti-head mAbs were combined (Fig. 5b). In contrast, when combinations including the highly bactericidal mAb 1C6 were tested, no synergy could be measured, possibly because the response was already at the plateau. Interestingly, the combination of the stalk-binding mAb 3H12 with mAbs targeting the head domain (2C4 and 5D11) elicited high hSBA titers, whereas a combination of this mAb with other mAbs targeting the stalk gave different results: synergy with mAb 7F11 and interference with mAb 10D12 (Fig. 5b).

Similarly to fHbp and NHBA, also in this case synergistic activity was observed between mAbs that were isolated either from the same or from different subjects.

**Figure 6:**
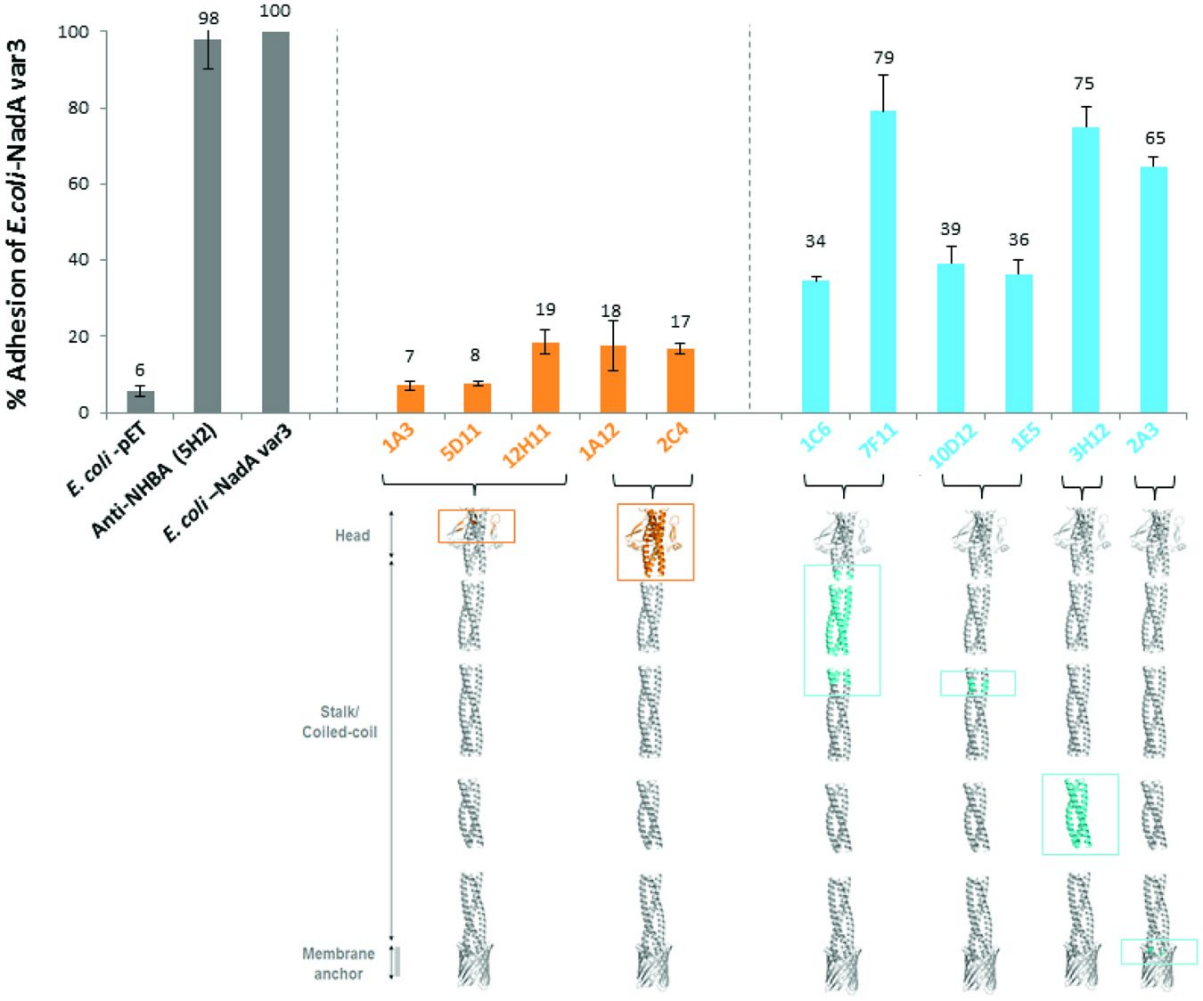
Inhibition by anti-NadA HuFabs of *E. coli*-NadA variant 3 adhesion to Chang epithelial cells. Controls grey bars: adhesion of *E. coli*-pET (Negative control), *E. coli*-NadA variant 3 pre-incubated with anti NHBA HuFab (unrelated antibody) and *E. coli*-NadA variant 3 (positive controls). Orange bars: adhesion of *E. coli*-NadA variant 3 pre-incubated with anti-NadA Head HuFabs. Blue bars: adhesion of *E. coli*-NadA variant 3 pre-incubated with anti-NadA Stalk HuFabs. Incubation of bacteria (MOI 100) with cells was performed at 37 °C for 3 h. The number of adherent bacteria is expressed as percentage of the value of the adhesive *E. coli*-NadA variant 3 bacteria counted at the end of the incubation period. Values represent the mean ± standard deviation of one representative experiment, out of three, performed in triplicate. Epitopes highlighted in NadA structure model^16^ at bottom of the graph.

NadA is a trimeric coiled coil adhesion, which is involved in meningococcal adhesion to human epithelial cells. To assess if anti-NadA antibodies could prevent bacterial adhesion, we performed an inhibition/adhesion assay. We have previously shown that surface expression of NadA in *E. coli* (*E. coli*-NadA) promotes bacterial adhesion to Chang conjunctival epithelial cells^29,30^. Deletion mutants lacking the N-terminal ‘head’ region of the molecule were unable to bind Chang cells, indicating that this protein domain is involved in receptor recognition^29^. To avoid protein structural problems induced by amino acid deletions, here we used a panel of monoclonal antibodies targeting different portions of NadA to refine the map of the region involved in specific cell binding. We pre-incubated *E. coli*-NadA with selected Fabs (Fig. 6) before proceeding with the infection scheme on Chang cell monolayers. Fabs were used instead of full length IgG to minimize an unspecific binding inhibition effect due to the steric hindrance of the Fc region of whole mAb. Assessment of the extent of infection was provided by the number of cell-associated colony-forming units (cfu) in cell lysates. The result of one representative experiment is shown in Fig. 6 in which 100% infection is the number of cfu obtained in absence of antibodies. The antibodies targeting the N-terminal moiety of the molecule elicited a 90% to 80% reduction of bacterial adhesion to cells whereas antibodies that recognize the stalk region only show a 20% to 60% reduction in adhesion; in the latter case a steric hindrance effect rather than an epitope masking effect cannot be excluded. Overall, these data suggest that the N-terminal head is the crucial region supporting the adhesion of the bacterium to epithelial cells.

## Discussion

In this work, we have reported for the first time a translational study exploring the human antibody response to each of three main recombinant protein antigens included in the 4CMenB vaccine against serogroup B *Neisseria meningitidis*. Monoclonal antibodies were obtained from plasmablasts of three vaccinees and extensively characterized both as Fab fragments and as full length IgG1 mAbs, thus providing a comprehensive overview on the quality of the antibody response and on the molecular mechanism of protection mediated by each single protein antigen.

Overall, the larger panel of antibodies was that obtained against NadA (128 different V_H_/V_L_ antibody sequence pairs), whereas the lower repertoire complexity was found for NHBA (only 25 V_H_/V_L_ antibody sequence pairs isolated from only 2 out of the 3 subjects), suggesting an intrinsically different immunogenicity of the three antigens. In all cases, the pattern of antigen recognition was quite heterogeneous, with mAbs generally targeting epitopes located on different regions of the proteins; thus indicating that the three vaccine components contain multiple protective epitopes. In a recently published paper, Beernink and coworkers^22^ focused their analysis on a subpanel of ten anti-fHbp antibody fragments (Fabs) from this study, and found that none of the Fabs was able to inhibit FH binding, thus suggesting that the FH binding site might not be accessible to the immune system upon immunization. Here we confirmed the previous results with the whole set of 12 Fabs and the 5 selected full length mAbs; however, we believe that the small number of monoclonal antibodies that are available in this panel does not permit generalized conclusions on whether or not the FH binding site might be at all accessible to antibody binding. As an extension of the present investigation, a longitudinal study is being performed analyzing the repertoire of anti-fHbp human mAbs isolated from three additional vaccine recipients at different time points following vaccine administration. The data obtained on the emerging panel of more than 100 anti-fHbp mAbs under investigation will be instrumental to confirm or dismiss this hypothesis.

Several papers have been published on the characterization of anti-fHbp murine mAbs^16,31–40^. Only in a few instances anti-fHbp mAbs were found to display serum bactericidal killing when tested alone using human serum as source of exogenous complement, whereas in most cases they were found to have synergistic activity and promoted hSBA killing when combined with a second mAb. As described previously, the density of the target antigen on the bacterial surface is fundamental for C3 deposition and complement activation^35^. Furthermore, antibody subclass is also important for functional activity, as it was shown that functionality of IgG3 > IgG1>>IgG2^36^. For this reason, in this work a high expressor prototype strain H44/76 expressing variant 1 fHbp was used for hSBA testing and all the recombinant mAbs were obtained as IgG1, as this is the most frequent subclass present in human serum. Moreover, a requisite that was generally considered to be important for an anti-fHbp mAb couple to become bactericidal is that at least one of antibodies targets the fH binding site^32,35^. Although none of the human mAbs included in this study targets the FH binding site, all the couples including mAbs targeting non-overlapping regions on fHbp are functional in hSBA, thus indicating that inhibition of FH binding is not crucial and that the density of the antigen-bound antibodies is the most important requirement for efficient complement activation and killing.

Interestingly, even if the vaccine contains only variant 1 fHbp, human recipients produced antibodies that were cross-reactive against all the three variants; most importantly, combination of the two cross-reactive mAbs 1G3 and 1A12 was bactericidal against strains expressing each of the three fHbp variants, thus indicating that polyclonal sera of subjects immunized with 4CMenB contain at least low abundance cross-protective antibodies.

The FHbp structure is characterized by an N-terminal taco-shaped barrel and a C-terminal eight stranded beta barrel. When anti-fHbp mAbs were screened on the protein chip, they recognized either the entire C-term beta barrel fragments or the full length protein, whereas none of the other fragments (either on N-term or C-term portion) was ever hybridized. The reason why N-terminal portions containing this domain are not recognized by Protein chip analysis could indicate some disorder in the recombinant fragments, which are unable to properly recapitulate the target regions of these specific mAbs independently from the C-terminal domain. This is the case of the cross reactive mAb 1G3. When the higher-resolution HDX-MS methodology was employed, using native proteins in solution, the results showed that the epitope was composed of two peptides located at the N-terminus of fHbp.

NHBA is also composed by two structural regions, a highly stable C-terminal beta barrel domain and a mostly unfolded N-terminal portion, mainly containing linear epitopes. Of the six anti-NHBA human mAbs included in this study, those targeting the C-term display the highest affinity whereas the two mAbs that recognize epitopes located on the N-term region are weak binders. Similarly to anti-fHbp mAbs, none of the anti-NHBA mAbs displays functional SBA activity when tested alone; however, even when a combination of two mAbs was tested, this was negative in the SBA activity using human complement, in line with previous reports showing that anti-NHBA antibodies were not functional in hSBA while still conferring passive protection from bacteremia in an infant rat model^28^. Interestingly, if rabbit serum was used in place of human serum as source of exogenous complement, then some combination of mAbs were positive in SBA; thus suggesting that factors specifically present in the human complement might interfere with the assay. It is known that human FH is one of the most abundant serum components and that it acts by down regulating the alternative complement cascade. Meningococcus specifically binds human FH - but not FH of other animal species - mainly through fHbp, thus protecting itself from complement mediated killing. In presence of human complement, FH would bind on fHbp thus rendering the bacterium more resistant to killing; in this framework of simultaneous complement down regulation and presence of a target antigen that is sparsely distributed on the bacterial surface, antibody binding to NHBA might not be sufficient to properly activate complement cascade and promote bacterial killing. It was previously reported that upon immunization with 4CMenB, a synergistic bactericidal activity could be exerted by anti-NHBA and anti-fHbp variant 1 human antibodies against strains expressing NHBA and a vaccine-mismatched fHbp variant 2^41^. A possible explanation would be that anti-fHbp variant 1 antisera might bind –even if with low affinity- to fHbp variant 2 thus competing with FH binding and ultimately reducing the threshold for effective bactericidal killing by anti-NHBA antibodies. In this study we have shown that immunization with fHbp variant 1 indeed leads to the production of mAbs able to cross react to variant 2 and variant 3, and this observation could further support the presented hypothesis. It will be interesting to perform studies using a combination of anti-NHBA and with anti-fHbp human mAbs to assess whether bactericidal activity can be restored in the presence of human FH.

Monoclonal antibodies isolated against NadA display broad variability in terms of region recognized, with half of them mapping on the head, and half targeting different portions of the stalk. Past work conducted on anti-fHbp monoclonal antibodies showed that even small differences in the epitope that was recognized could have a big impact on the functional properties of the mAbs^32,34^. Here, we show that although eight out of the ten mAbs that recognize the head display a similar epitope mapping profile according to Protein chip analysis (region 52–72), they show different affinities to the protein, and two of them are not able to bind either the recombinant or the native NadA protein. Furthermore, mAbs 12H11 and 5D11 display different ability to elicit bactericidal response, with hSBA titers of 64 and 2,048, respectively, thus confirming previous observations with anti-fHbp mAbs.

In contrast to what was observed for anti-fHbp and anti-NHBA mAbs, anti-NadA antibodies were able to provide high and consistent bactericidal titers also when tested singularly against the high expressor 5/99 meningococcal strain. From previous studies it is known that, although the density of fHbp can vary quite substantially across different bacterial strains, this antigen is evenly distributed on the bacterial surface^42^. Nonetheless, in most of the cases SBA titers increased when mAbs were tested in combination. In contrast, NadA appears to be expressed in small clusters of protein trimers, and this organization would facilitate the juxtaposition of mAbs targeting flanking NadA molecules, which could co-operate to elicit high SBA titers. Previous papers had described NadA as an adhesin implicated in the binding and invasion of human epithelial cells, and had anticipated the key role of the head domain for full functional activity^29,43–46^. Here for the first time we show that only monoclonal antibodies targeting the head domain of NadA are capable to inhibit binding to human cells, thus ultimately confirming the presence of the cell binding domain within this portion of the molecule. This result has important implications from the vaccine perspective, as it demonstrates that NadA immunization can lead to the production of antibodies not only able to promote NadA-mediated killing of meningococcal strains, but also likely able to prevent initial bacterial colonization. Although clinical investigations carried out so far have not been able to demonstrate a clear impact of 4CMenB vaccination on MenB carriage, this is probably due to the low sample size and the relatively low number of events^46^; indeed, a trend toward lower acquisition of MenB and a significant 27% reduction in acquisition of other serogroups was reported by Read and colleagues^45^, however further studies are needed to confirm these preliminary results. Overall, the data presented in this paper reveal a remarkable synergistic effect between monoclonal antibodies –either isolated from the same or from different vaccinees- that recognize multiple protective epitopes on the same protein antigen, These results expand our knowledge on the functional role of 4CMenB antigens in meningococcal pathogenesis and provide us with further insights on the molecular mechanisms at the basis of the human protective response induced by 4CMenB vaccine.

## Materials and Methods

### Human samples

Human sampes obtained from adults immunization in a Phase I clinical study conducted in Krakow, Poland and sponsored by Novartis Vaccine, now part of the GSK group of Companies, using two doses of multicomponent serogroup B meningococcal vaccines containing recombinant fHbp ID 1. The Clinical trial protocol was approved by the Bioethics Committee of the District Medical Doctors’ Chamber in Krakow and the study was conducted in accordance with the Declaration of Helsinki. Written informed consent was obtained from each of the subjects.

### Recombinant Fabs production

H and L chain V region genes of single plasmablasts isolated from peripheral blood were amplified separately and then joined by overlap extension PCR. In particular, the heavy (H) and light (L) chain immunoglobulin variable (V) region genes were isolated from individual peripheral blood plasmablasts of three adult vaccines and cloned for recombinant production^22^. Products were cloned into pET22 as bicistronic expression cassettes encoding for Fab antibody fragments and sequenced^22^. Fab clones encoded by unique germ line were expressed in *Escherichia coli* Rosetta2 (DE3) strain (Novagen) in Enpresso B medium (Biosilta), according to manufacturer’s instructions. Induction was carried out with 1 mM IPTG for 24 h at 25 °C. Total cell lysis was performed in CelLytic Express (Sigma-Aldrich). Recombinant Fabs were purified by Ni^2+^-affinity chromatography (Ni-NTA agarose resin, Qiagen), buffer-exchanged to Tris-HCl 20 mM, NaCl 300 mM, sodium azide 0.05%, pH8 and stored at 4 °C. Recombinant Fabs were quantified by Bradford protein assay (Sigma-Aldrich) and their purity was assessed by SDS-PAGE after coomassie staining in non-reducing conditions.

### Recombinant mAbs production

Gene fragments, optimized for mammalian expression, corresponding to VH and VL of desired human monoclonal antibodies were synthetized by GeneArt (Thermo Fisher Scientific) by adding at the gene extremities the Eco31I site and an appropriate linker for single step cloning. Synthetic DNA strings were suspended with 50 μl nuclease-free water and digested with Eco31I restriction enzyme. Digested and purified DNA products were ligated into human pRS5a Igγ1, Igκ and Igλ expression vectors (NIBR) containing a human Ig gene signal peptide sequence and the Eco31I cloning site upstream of the human IgG1, Igκ or Igλ constant regions. Cloning was performed in *E. coli* DH5a cells, using standard ligation protocol. In the pRS5a antibody expression vectors, the transcription is under the CMV promoter and positive clones were selected based on resistance to ampicillin.

Transient production of recombinant antibodies in suspension Expi293 cells (Thermo Fisher Scientific) was performed according to manufacturer’s protocol. Equal amounts (15 μg each/30 ml of transfection volume) of IgH and corresponding IgL chain expression vector DNA were used to transfect Expi293 cells. Cells were harvested and supernatants collected 3 and 6 days after transfection.

To remove cell debris, culture supernatants were centrifuged at 900 × g for 10 min, filtered through a 0.22 µm pore size filter (Millipore) and dialyzed against sodium phosphate 20 mM pH 7. Recombinant antibodies were purified with Protein G beads (GE Healthcare) according to the manufacturer’s instructions. Antibodies were eluted with 0.1 M glycine (pH 3.0), in tubes containing 1 M Tris (pH 9.0). Buffer was exchanged to PBS. Recombinant antibodies were quantified by absorbance at 280 nm and their purity was assessed by SDS-page electrophoresis and Coomassie staining.

### Affinity ranking of Fabs by Gyros

The ranking of affinity was done on the basis of the W score. All Fabs were run by Gyrolab in dose-response curves to select the range of linearity of the response. Then Fabs were run at a single concentration within the range of linearity in 7 replicates to calculate the W value and standard deviation (for an extensive explanation of the method refers to (PCT/EP2014/066445; WO2015014922).

As capture reagents, biotinylated recombinant antigens (MenB antigens fHbp-variant 1.1, NHBA P2 and NadA variant 3) were used at 100 µg/ml (diluted in PBS/Tween20 0,01%). Antigens were biotinylated using EZ-Link sulfo-NHS-LC-Biotin (Thermo Scientific) at a molar excess of 10 moles of biotin: 1 mole of protein. As detection reagent, Goat Anti-Human IgG, Fab fragment specific-Alexa 647 (Jackson Immuresearch) were used at 25 nM. Dilutions were done in Rexipp F buffer (Gyros).

Wash station and pump solutions consisted of PBS with 0.01% Tween 20 (Sigma). All Fabs were analyzed using Gyrolab Bioaffy 200 CDs (Gyros) and the standard Gyrolab three-step method (capture-analyte-detection).

### Epitope mapping by Protein chip and Pep Scan

Preliminary epitope mapping studies were performed by a combination of techniques including Protein and Peptide microarrays. In particular, human Fabs and mAbs were tested on protein microarrays containing recombinant full length proteins as well as overlapping fragments of heterogeneous length (40aa to 602aa) spanning the entire protein sequences of fHbp variant 1.1, variant 2.16 and variant 3.28, NHBA peptide variant 2^24^, and NadA variant 3^25^. In addition, full-length NHBA peptide var 3, 17, 20 and 21 were spotted on Protein chip to verify cross-reactivity. Ultimately, the binding profile of each Fab to the antigens’ fragments spotted on the microarray defined the minimal common portion of the protein implicated in the interaction. Briefly, recombinant antigens were spotted on nitrocellulose-coated slides (FAST slides, Maine Manufacturing) using the no-contact Marathon Spotter (Arrayjet, Edinburgh, UK).

Differently, peptide microarrays for PepScan analysis were produced using synthetic 15-mer overlapping peptides with an offset of 4 amino acids covering the entire sequence of different variants of the three 4CMenB antigens. In particular, NHBA specific peptide microarrays contains a panel of 561 peptides representing the complete sequence of 10 different NHBA variants (P1, P2, P3, P5, P10, P17, P18, P20, P21 and P20)^24^, NadA specific peptide microarrays contains a panel of 348 peptides covering 5 NadA variants (variant 1, 2, 3, 4 and 5)^25^ and finally fHbp specific peptide microarrays contains a panel of 363 peptides of 12 different fHbp variants (1.1, 1.4, 1.13, 1.14, 1.15, 2.16, 2.19, 2.25, 2.77, 3.28, 3.45, NL10A). Chemically synthetized peptides were immobilized on glass slides via a flexible linker generating microarrays displaying directed and covalently attached peptides (JPT Technologies GmbH, Berlin, Germany).

Nonspecific binding was minimized by preincubating protein or peptide microarray slides with a blocking solution (BlockIt, ArrayIt) for 1 hour. *E. coli* Fabs and mAbs were diluted 1:50 and 1:2000 respectively in BlockIt and overlaid on the protein arrays for 1 h at room temperature; mAbs were diluted 1:200 in BlockIt and overlaid on the peptide arrays for 1 h at room temperature. AlexaFluor®647-conjugated anti-Human IgG secondary antibody (Jackson Immunoresearch) was added for 1 h at room temperature in the dark, before proceeding with slide scanning. Fluorescence signals were detected by using a PowerScanner confocal laser scanner (Tecan Trading AG, Switzerland) and the 16-bit images were generated with PowerScanner software variant 1.2 at 10 μm/pixel resolution and processed using ImaGene 9.0 software (Biodiscovery Inc, CA). Elaboration and analysis of image raw Fluorescence Intensity (FI) data was performed using in-house developed software and R scripts. Signals were considered as positive when their MFI value was higher than 15,000, corresponding to the MFI of protein spots after detection with anti-AlexaFluor®647 polyclonal antibody (Jackson Immunoresearch) alone, plus 10 standard deviation values. The peptide and protein microarray data have been deposited to National Center for Biotechnology Information’s Gene Expression Omnibus database (http://www.ncbi.nlm.nih.gov/geo/query/acc.cgi) under series accession number GSE98883.

### Epitope mapping by HDX-MS analysis

The antibody/antigen complexes were formed by mixing 54 pmol of the selected antibody to an equimolar amount of recombinant fHbp var.1 or NHBA p2, incubated for 30 min at room temperature and HDX-MS analysis were performed as previously reported^32^. A control experiment for each complex was performed with the antigen alone at the same condition previously described, using PBS instead of the antibody.

### Surface Plasmon Resonance

SPR was used to measure and compare the binding affinities of the tested antigens to the monoclonal antibodies under study. All SPR experiments were performed using a Biacore T200 instrument at 25 °C (GE Healthcare). For the single-cycle kinetics (SCK) experiments, the latter well-suited for the measurement of high affinity binding events, a commercially available Human Fab Antibody Capture Kit (GE Healthcare) was used to immobilize an anti-human Fab antibody by amine coupling on a carboxymethylated dextran sensor chip (CM-5; GE Healthcare). Experimental running buffer contained 10 mM Hepes, 150 mM NaCl, 3 mM EDTA, 0.05% (vol/vol) P20 surfactant, pH 7.4. A density level yielding ∼8,000–10000 response units (RUs) was prepared for the immobilization on two flow cells of the CM5 chip. The anti-human IgG were used then to capture between ∼800–1200 RU of the tested mAbs at a concentration of 10–20 μg/ml in running buffer. For the determination of K_D_ and kinetic parameters, a titration series of five consecutive injections of increasing analyte concentration (different ranges used according to antigen-antibody pair were: fHbp variant 1.1 0.78–12.5 nM, fHbp variant 2.16 1.56–25 nM, fHbp variant 3.28 0.3–5 nM and 3.1–50 nM; NHBA p2 3.1–50 nM; NadA variant 3 0.39–6.25 nM, 0.78–12.5 nM, 3.125–50 nM) at a flow rate of either 30 or 40 μL/min, followed by a single final surface regeneration step with buffer containing 10 mM glycine pH 2.1 (180 seconds, flow rate of 10 μL/min) was performed using the standard SCK method^47^ implemented by the Biacore T200 Control Software (GE Healthcare). Anti-human antibody-coated surfaces without captured mAb were used as the reference channel. A blank injection of buffer only was subtracted from each curve, and reference sensorgrams were subtracted from experimental sensorgrams to yield curves representing specific binding. The data shown are representative of two independent experiments. SPR data were analyzed using the Biacore T200 Evaluation software (GE Healthcare). Each sensogram was fitted with the 1:1 Langmuir binding model, including a term to account for potential mass transfer, to obtain the individual k_on_ and k_off_ kinetic constants; the individual values were then combined to derive the single averaged K_D_ values reported.

For testing competition between antibody pairs, mAbs (1000 RU) were immobilized individually on a CM5 sensor chip as described above. In the following step, 100 RU of fHbP were captured on the immobilized mAbs by injecting a 100 nM solution in running buffer, resulting in the formation of stable mAb:fHbP complexes on the chip surface. The final step consisted in injecting the second mAb (100 nM in running buffer) to be tested. If the two antibodies are compatible, (i.e. occupying spatially separated epitopes) an increase in the SPR signal is observed, otherwise, the SPR signal remains virtually unchanged, indicating mutual exclusion of the two antibodies tested.

### Bacterial strains and culture conditions

*N. meningitidis* strains M4407 and mutant strain 5/99ΔΔ CP2 [5/99 strain containing a marker-free deletion of *nadA* and *nhba* genes and where NHBA P2 expression was obtained by *ex-locus* complementation of *nhba* gene under the control of IPTG-inducible tac promoter, as previously described]^11^ were selected as reference strains for NHBA; NMB, DE11445 and 5/99 were used as reference strains for NadA variant 3, variant 2 and variant 1 respectively. MC58 and H44/76 were used as reference strains for fHpb variant 1, M08–240104 (UK104) and M01-0240320 (UK320) were used as reference strains for fHbp variant 2 and variant 3 respectively. Bacteria were grown on chocolate agar (Biomerieux) at 37 °C, 5% CO2 overnight. For liquid cultures, colonies from overnight growth were used to inoculate 7 ml cultures (in MH broth supplemented with 0.25% glucose) to an optical density at 600 nm (OD_600_) of 0.05. The culture was incubated for approximately 1.5 to 2.5 h at 37 °C with shaking until early log (OD_600_ of 0.25) or mid-log phase (OD_600_ of 0.5).

### Flow cytometry analysis

The ability of Fabs to bind antigen exposed on the surface of *N. meningitidis* bacteria was determined using a FACScan flow cytometer. Bacteria grown until mid-log phase (OD_600_ of ~0.5) were incubated with monoclonal antibodies at the concentration of 50 ug/ml. Antibody binding was detected using an Anti-Human IgG (Fab specific)-FITC conjugated produced in goat (Sigma) or an Anti-Human IgG-FITC conjugated produced in goat (Jackson Immuno Research) at a 1:100 dilution. Bacteria plus PBS-1%BSA and secondary antibody were used as negative control.

### Serum bactericidal activity assay

Serum bactericidal activity against *N. meningitidis* strains was evaluated as reported elsewhere^27,48^. Bacteria grown until early log phase (OD_600_ of ~0.25) were diluted in Dulbecco’s Phosphate Buffered Saline (DPBS) containing 1% bovine serum albumin (BSA) and 0.1% glucose at the working dilution of 10^4^–10^5^ and incubated with serial two fold dilutions of test monoclonal antibodies starting from a concentration of 125 µg/ml. Serum bactericidal titers were defined as the monoclonal antibody dilution resulting in 50% decrease in CFU per milliliter after a 60-min incubation of bacteria with the reaction mixture compared to the control CFU per milliliter at time zero. Pooled baby rabbit sera from Cedarlane or human serum, obtained from volunteer donors under informed consent, have been used as a complement source for rSBA or hSBA respectively.

### Binding inhibition assays

*Escherichia coli* BL21(DE3) (New England Biolabs) was used to express full-length NadA variant 3 following transformation with pET21-*nadA*, as previously described^29^, or the empty vector as control. *E. coli* was cultured at 37 °C in Luria–Bertani (LB) broth supplemented with 100 μg/mL of ampicillin. Protein expression for full-length NadA was achieved without addition of IPTG exploiting the expression due to the leakage in the induction system. Chang epithelial cells (Wong-Kilbourne derivative, clone 1-5c-4, human conjunctiva, ATCC CCL-20.2) were maintained in Dulbecco’s modified Eagle’s medium (DMEM) supplemented with antibiotics and 10% heat-inactivated fetal bovine serum (FBSi). Cells were grown at 37 °C in 5% CO2.

Chang cells were seeded in a 24 well plate (1.5 × 10^5^ cells/well) in antibiotics-free medium with 1% iFBS (Infection Medium) and were allowed to grow for 24 h. For adhesion analysis, *E. coli* wild type or expressing NadA were added at a multiplicity of infection^49^ of 100 and allowed to adhere for 3 h at 37 °C and 5% CO_2_. Unattached *E. coli* were removed by repeated washing, and bacteria were counted using serial dilution plating. For inhibition of binding assay, recombinant *E. coli* wild type or expressing NadA were pre-incubated in rotation with 200 ng/μL HuFab or 70 ng/μL mAb or infection medium for 1 hour at 4 °C before addition to the cells.

### Data availability

All data generated or analysed during this study are included in this published article (and its Supplementary Information files).

The datasets generated during Protein chip and PepScan and analysed during the current study are available in the Gene Expression Omnibus database (GEO; http://www.ncbi.nlm.nih.gov/geo/query/acc.cgi) under series accession number GSE98883.

## Electronic supplementary material

Supplementary material
