# Supplementary material for: Human protective response induced by meningococcus B vaccine is mediated by the synergy of multiple bactericidal epitopes
Source: Sci Rep. 2018 Feb 27;8:3700. doi: 10.1038/s41598-018-22057-7 (PMC5829249; doi:10.1038/s41598-018-22057-7)
Supplement: Supplementary file 1 — Supplementary material [file 41598_2018_22057_MOESM1_ESM.pdf]

Human protective response induced by meningococcus B vaccine is mediated by the synergy of multiple bactericidal epitopes

M. Giuliani[1§], E. Bartolini[1§], B. Galli[1], L. Santini[1], P. Lo Surdo[1], F. Buricchi[1], M. Bruttini[1,2], B. Benucci[1,2], N. Pacchiani[1], L. Alleri[1], D. Donnarumma[1], W. Pansegrau[1], I. Peschiera[3], I. Ferlenghi[1], R. Cozzi[1], N. Norais[1], M. M. Giuliani[1], D. Maione[1], M. Pizza[1], R. Rappuoli[1], O. Finco[1] and V. Masignani[1*]

[1] GSK, Siena, Italy

[2] University of Siena

[3] University of Bologna

[§] Equal contributors

[*] Corresponding author

Supplementary material

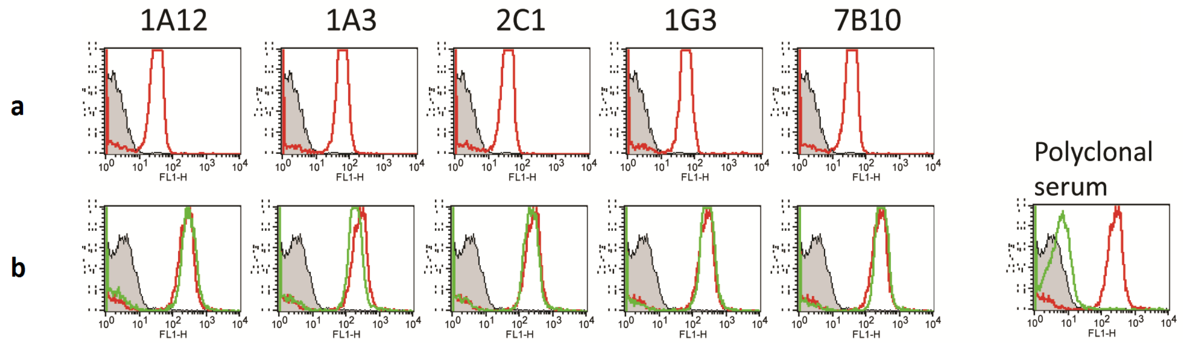

Figure S1. Flow cytometry analysis of hfH binding inhibition. (a): histograms showing binding to live meningococci of group B strain MC58 when incubated with 50ug/ml of HumAbs anti-fHbp (red line). Gray-filled histogram represents negative control, bacteria incubated with PBS and anti-human IgG FITC-conjugated. **(b):** histograms showing effect of HumAbs anti-fHbp on binding of human fH to live meningococci of strain MC58. Red line, binding of hfH alone (10µg/ml); green line, binding of hfH when bacteria were pre-incubated with 50 ug/ml of HumAbs anti-fHbp. None of the IgG1 mAbs were able to inhibit hfH binding. In the last histogram of panel B, inhibition of hfH binding by murine anti-fHbp polyclonal serum (1:100). Gray-filled histogram represents negative control bacteria incubated with PBS and secondary antibodies FITC-conjugated antibodies.

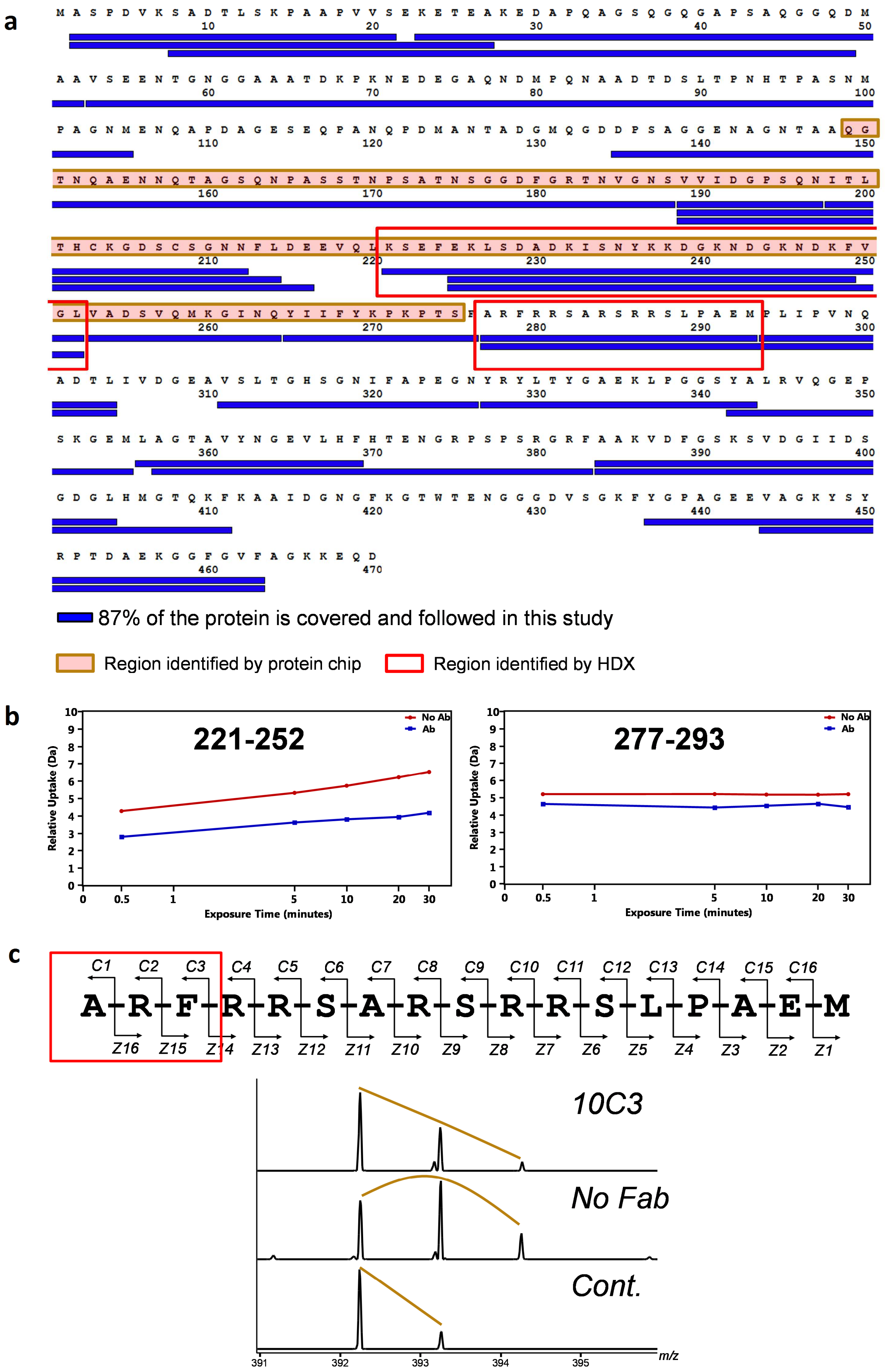
a
M A S P D V K S A D T L S K P A A P V V S E K E T E A K E D A P Q A G S Q G Q G A P S A Q G G Q D M
A A V S E E N T G N G G A A A T D K P K N E D E G A Q N D M P Q N A A D T D S L T P N H T P A S N M
P A G N M E N Q A P D A G E S E Q P A N Q P D M A N T A D G M Q G D D P S A G G E N A G N T A A Q G
T N Q A E N N Q T A G S Q N P A S S T N P S A T N S G G D F G R T N V G N S V V I D G P S Q N I T L
T H C K G D S C S G N N F L D E E V Q L K S E F E K L S D A D K I S N Y K K D G K N D G K N D K F V
G L V A D S V Q M K G I N Q Y I I F Y K P K P T S F A R F R R S A R S R R S L P A E M P L I P V N Q
A D T L I V D G E A V S L T G H S G N I F A P E G N Y R Y L T Y G A E K L P G G S Y A L R V Q G E P
S K G E M L A G T A V Y N G E V L H F H T E N G R P S P S R G R F A A K V D F G S K S V D G I I D S
G D G L H M G T Q K F K A A I D G N G F K G T W T E N G G G D V S G K F Y G P A G E E V A G K Y S Y
R P T D A E K G G F G V F A G K K E Q D
87% of the protein is covered and followed in this study
Region identified by protein chip
Region identified by HDX
b
221-252
277-293
No Ab
Ab
Relative Uptake (Da)
Exposure Time (minutes)
c
A R F R R S A R S R R S L P A E M
C1 C2 C3 C4 C5 C6 C7 C8 C9 C10 C11 C12 C13 C14 C15 C16
Z16 Z15 Z14 Z13 Z12 Z11 Z10 Z9 Z8 Z7 Z6 Z5 Z4 Z3 Z2 Z1
10C3
No Fab
Cont.
m/z

Fig. S2. Epitope mapping of the NHBA P2 region targeted by HumAb10C3. **a**. Sequence of the NHBA P2 where the protein portion identified by Protein scan is evidenced in pink (Table 2b); and peptides followed in the HDX-MS study are underlined in blue. They represent 87% of the protein sequence coverage. Peptides presenting a difference of deuterium incorporation when the antigen is bound to the HumAb10C3 are boxed in red. **b**. HDX-MS plots of the peptides showing a difference in deuterium uptake in presence and in absence of HumAb10C3 (in red and blue, respectively). The peptide 221-252 is included in the sequence identified by Protein chip while the peptide 277-293 is successive to this sequence. **c**. ETD fragmentation of the peptide 277-293 pinpoints that the deuterium uptake is located on the first 3 amino acids as deduced from the isotopic distribution of the c3 fragment. As deduced by the Protein chip analysis these residues are not indispensable for the HumAb binding.

Supplementary Materials and Methods

Inhibition of binding of fH

The ability of the HumAbs to inhibit binding of fH to live bacteria of MC58 strain was measured by flow cytometry. For competitive inhibition analysis of fH binding, the bacterial cells were grown, harvested, and resuspended in PBS-1%BSA buffer as described above for measuring mAb binding to *N. meningitidis* by flow cytometry. Bacteria were incubated with anti-fHbp mAb (50 μg/ml in PBS-1%BSA buffer) for 30 min at room temperature, followed by the addition of purified human fH (10 μg/ml), which was incubated for an additional 30 min at room temperature in a final reaction volume of 100 μl. fH binding was detected with a goat polyclonal antiserum to fH (Calbiochem) diluted 1:200 and incubated for 30 min at room temperature, followed by additional 30 min incubation with a donkey anti-goat IgG–fluoresceinisothiocyanate (FITC) conjugate (Jackson Immunoresearch) diluted 1:100 in PBS-1%BSA buffer.
